# The Tumor Suppressor Gene Retinoblastoma-1 Is Required for Retinotectal Development and Visual Function in Zebrafish

**DOI:** 10.1371/journal.pgen.1003106

**Published:** 2012-11-29

**Authors:** Michael Gyda, Marc Wolman, Kristin Lorent, Michael Granato

**Affiliations:** Department of Cell and Developmental Biology, University of Pennsylvania Perelman School of Medicine, Philadelphia, Pennsylvania, United States of America; Medical College of Wisconsin, United States of America

## Abstract

Mutations in the retinoblastoma tumor suppressor gene (*rb1*) cause both sporadic and familial forms of childhood retinoblastoma. Despite its clinical relevance, the roles of *rb1* during normal retinotectal development and function are not well understood. We have identified mutations in the zebrafish *space cadet* locus that lead to a premature truncation of the *rb1* gene, identical to known mutations in sporadic and familial forms of retinoblastoma. In wild-type embryos, axons of early born retinal ganglion cells (RGC) pioneer the retinotectal tract to guide later born RGC axons. In *rb1* deficient embryos, these early born RGCs show a delay in cell cycle exit, causing a transient deficit of differentiated RGCs. As a result, later born mutant RGC axons initially fail to exit the retina, resulting in optic nerve hypoplasia. A significant fraction of mutant RGC axons eventually exit the retina, but then frequently project to the incorrect optic tectum. Although *rb1* mutants eventually establish basic retinotectal connectivity, behavioral analysis reveals that mutants exhibit deficits in distinct, visually guided behaviors. Thus, our analysis of zebrafish *rb1* mutants reveals a previously unknown yet critical role for *rb1* during retinotectal tract development and visual function.

## Author Summary

Before an organism can execute necessary behavioral responses to environmental stimuli, the underlying neural circuits that regulate these behaviors must be precisely wired during embryonic development. A properly wired neural circuit is the product of a sophisticated collaboration of multiple genetic pathways that orchestrate cell type specification, the extension and growth of the cell processes that connect each circuit component, and the refinement of these connections. In an unbiased genetic screen designed to identify the genes required for proper circuit formation in developing zebrafish embryos, we identified a human disease causing mutation in the *retinoblastoma-1 (rb1)* gene that disrupts the formation of the zebrafish visual circuit. *rb1* canonically functions to regulate the cell cycle, and when mutated the loss of *rb1*-mediated cell cycle control elicits childhood ocular tumor formation. Genetic models of *rb1* have been developed to study the developmental role of *rb1* in the retina; however, ectopic cell proliferation and death within the retina have largely precluded the ability to evaluate the formation and integrity of neural circuits connecting the retina with the brain. In this study, through genetic and cellular analysis of a zebrafish *rb1* mutant, we reveal a novel role for *rb1* in regulating the establishment and functionality of the visual circuitry.

## Introduction

Biallelic mutations in the retinoblastoma susceptibility gene *rb1* are causal for intraocular childhood retinoblastomas. Rb1 is a member of a gene family that consists of three members, p105/Rb1, p107/Rb-like1, and p130/Rb-like2, collectively known as “pocket proteins” [1]. The activity of these proteins is controlled, in part, by cyclin/cyclin-dependant kinase complexes. Upon activation, Rb proteins bind to an array of proteins, including members of the E2F family of transcription factors to execute a range of cellular functions, including cell cycle exit, terminal differentiation, and cortical cell migration [2]. In humans, germline or somatic mutations occur throughout the 180 kb genomic region spanning the *rb1* gene, including its promoter region, exons, and intronic essential splice sites, resulting in bilateral or unilateral retinoblastomas within the first 2 years of life [3], [4].

Given its clinical relevance, the role of Rb1 during embryonic development and during tumor suppression has been studied intensely, mostly using mouse models [5]. Rb1 is expressed ubiquitously during murine development, postnatally, and continues to be expressed in adults. Embryos harboring non-conditional Rb1 knockout alleles exhibit ectopic proliferation and apoptosis throughout the nervous system and die prenatally at embryonic day 14.5 [6], [7], [8]. Embryos with conditional loss of Rb1 in the retina display ectopic division and considerable apoptosis of retinal transition cells starting at E10 [9], [10], [11], [12]. Retinas in these animals contain reduced numbers of rods, bipolar cells, and RGCs, yielding a retina with a thin outer nuclear layer and a hypoplastic optic nerve. However, the etiology of optic nerve hypoplasia and if/how Rb1 functions in RGC axonal guidance has not been examined. Similarly, electroretinogram recordings from Rb1 deficient mouse retinas have revealed reduced photoreceptor to bipolar to amacrine signal transmission [10], yet the behavioral consequences have not been examined.

Here, we report that zebrafish *space cadet* mutants carry a *rb1* mutation found in cases of sporadic and familial human retinoblastoma [13], [14], [15], [16]. In zebrafish *rb1* mutants, RGC precursors show delayed exit from the cell cycle and hence a delay in the generation of early-born, postmitotic RGCs, whose axons are critical for pioneering the retinotectal tract. This delay leads to RGC intrinsic axon guidance defects, aberrant retinotectal connectivity, and deficits in phototactic behaviors. Together, this work describes a novel model for understanding the developmental role of *rb1* and reveals a previously unknown role for *rb1* in the formation of the retinotectal tract.

## Results

### *space cadet* harbors a disease causing mutation in *rb1*

**Figure 1:**
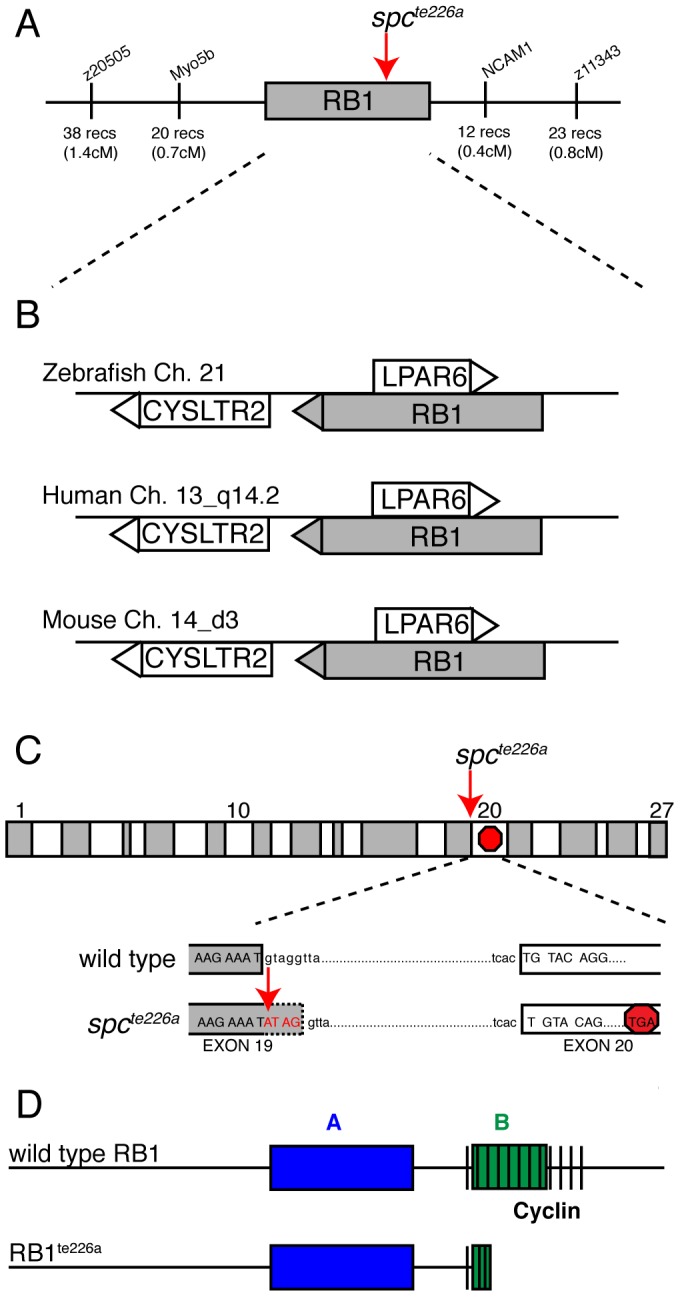
*spc^te226a^* mutation induces a premature stop codon and truncation of Rb1. (A) Recombination mapping places the *spc^te226a^* mutant locus within a 1.1 cM interval on chromosome 21 that shows syntenic conservation (B) of *rb1, lpar6,* and *cysltr2* transcripts with human chromosome 13_q14.2 and mouse chromosome 14_d3. (C) *rb1* exons depicted as alternating gray/white blocks. *spc^te226a^* mutation at splice donor site of intron 19 (nt1912+1) causes frameshift and premature stop codon in exon 20. (D) Estimated Rb1^te226a^ protein product with truncated B- and cyclin-domains.

We previously identified two mutant *space cadet* alleles, based on abnormal startle response behavior to acoustic or tactile stimuli [17], [18]. Using recombination mapping, we mapped the *space cadet^te226a^* allele to a 1.1 cM interval on chromosome 21 between single nucleotide polymorphic markers in the *myo5b* locus (20 recombinants/2688 meioses), and in the *ncam1* locus (12 recombinants/2688 meioses), respectively (Figure 1A). This genomic interval contains several annotated genes, including *rb1, lpar6, and cystlr2*, which have retained syntenic positional conservation between humans, mice and zebrafish (Figure 1B). Sequencing of *rb1* cDNAs isolated from *spc^te226a^* larvae revealed the presence of 4 nucleotides inserted between exon 19 and exon 20. Subsequent sequencing of genomic DNA isolated from *spc^te226a^* larvae confirmed a single nucleotide change in the splice donor sequence of intron 19 (nt1912+1: G to A; Figure 1C). This generates a cryptic splice site donor, resulting in the 4 base pair insertion into the *rb1* mRNA. This 4 base pair insertion causes a premature stop codon in exon 20, predicted to truncate the protein at amino acid 677, thereby severely truncating the B domain and cyclin domain essential for Rb1 function (Figure 1D) [15]. Interestingly, identical mutations have been reported in human patients with familial and sporadic forms of retinoblastoma [13], [14], [15], [16].

The zebrafish *rb1* gene is 67% similar (52% identical-based on amino acid sequence) to the mouse and human *rb1*. The Rb1 protein consists of an A and B domain forming Rb1's binding “pocket”, and a cyclin binding domain (Figure 1D), and shows 81% amino acid similarity (66% identical) between zebrafish and mammalian *rb1* in these critical domains. Thus, sequence homology, syntenic conservation, and cDNA sequence analysis provide compelling evidence that *space cadet* phenotypes are caused by an *rb1* gene mutation known to cause retinoblastoma. Sequence analysis of the second mutant *space cadet^ty85d^* allele did not reveal any changes in the coding sequence or in any of the splice donor and acceptor sites, suggesting that this allele is caused by a regulatory mutation in the *rb1* locus. Importantly, analyses of *spc^te226a^* and *spc^ty85d^* mutants revealed no significant differences with regards to the strength of the phenotypes examined below (Table 1). From here on, we will refer to the *space cadet* gene as *rb1* and describe anatomical and behavioral defects in the *rb1^te226a^* allele.

**Table 1:** Comparison of the retinal phenotypes observed at 36 hpf in the two *rb1* mutant alleles.

Genotype	Retinas with complete RGC axon exit defects	^#^ Mean M-phase nuclei +/− SEM per retina (n = number of retinas examined)
*rb1^te226a/+^*	0/20 (0%)	85.45+/−3.22 (20)
*rb1^te226a/te226a^*	26/30 (86%)	42.2+/−4.7 (15)
*rb1^te226a/ty85d^*	*13/18 (72%)	34+/−2.9 (13)

* *rb1^te226/ty85d^* embryos were generated from a cross between *spc^ty85d^* heterozygotes and *rb1^te226a^* heterozygotes, and then genotyped for the presence of the *rb1^te226a^* allele. Of the genotyped *rb1^te226a^* heterozygotes, only half are expected to carry the *spc^ty85d^* allele, thus reducing the total number of mutant retinas by half, and numbers for *rb1^te226/ty85d^* were adjusted accordingly.

### *rb1* mutant embryos show delayed RGC axon outgrowth and reduced tectal innervation

**Figure 2:**
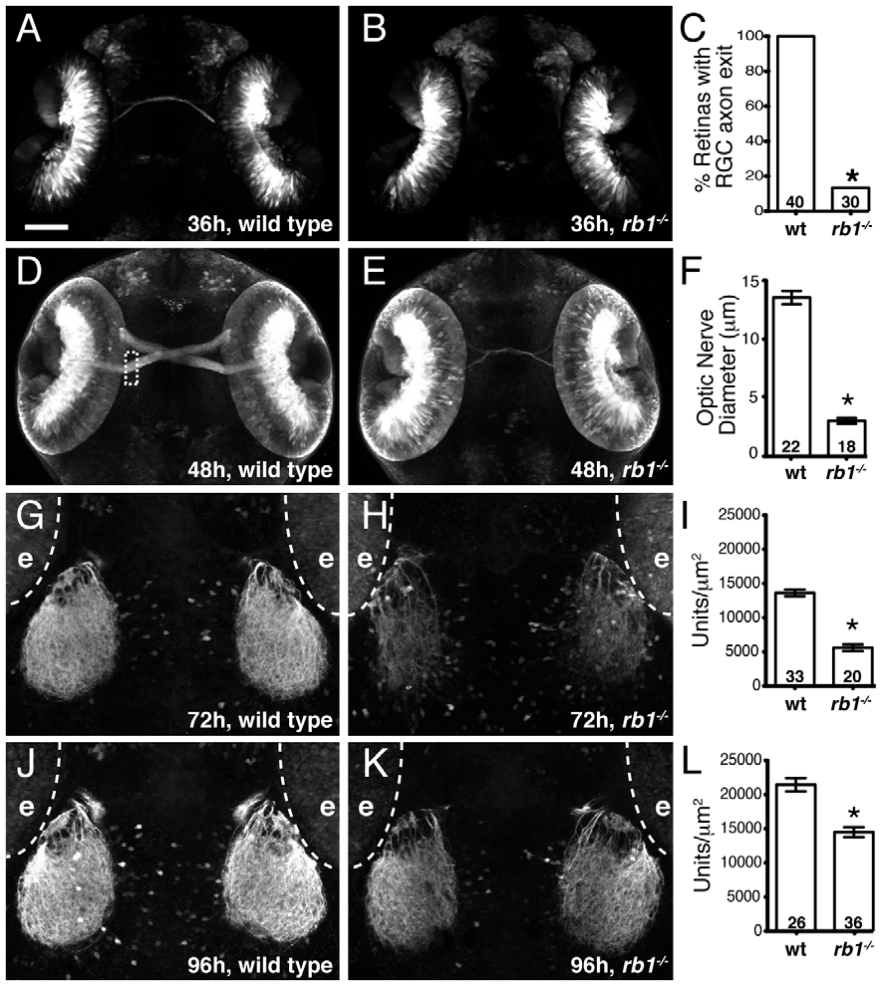
Rb1-deficient embryos possess delayed retinotectal projection. Dorsal view of maximum intensity projection of confocal z-stacks of RGC axons in wild type or *rb1^te226^; ath5:gfp^+/−^* embryos. (A–C) RGC axons fail to exit *rb1^te226^* retina at 36 hpf. (D–F) *rb1^te226^* optic nerves are hypoplastic. Diameter measured upon retinal exit, marked by dashed rectangle. (G–L) Tectal innervation is reduced in *rb1^te226^* at 72 (G–I) and 96 hpf (J–L). Innervation measured as a function of pixel intensity units per µm^2^ from summation intensity projections of tectal area (not shown, see Experimental Procedures). Digitally excluded eyes (e) are outlined by dotted line. (C, F, I, L) Bar graphs represent the mean with error bars denoting SEM. *p<0.001; one-way ANOVA (F, I, L) or binomial z-test (C). N retinas, optic nerves, or tecta shown at base of bar graphs. Anterior to the top of panel, scale bar = 50 µm.

During zebrafish embryogenesis, the earliest born RGCs begin extending axons at 32 hpf, cross the ventral midline of the diencephalon to form the optic chiasm at 36 hpf, and project dorsally to the contralateral optic tectum by 48 h to form a retinotectal pathway critical for mediating visually guided behaviors by 120 hpf [19]. We previously reported that 120 hpf stage *rb1* mutants display wild type like retinal lamination and expression of terminal RGC cell differentiation markers, but exhibit various RGC axonal pathfinding defects [18]. To determine the temporal onset and spatial site of RGC pathfinding errors in *rb1* mutant embryos, we used the *ath5:gfp* transgenic line expressed in RGCs to examine the development of the retinotectal trajectory [20]. Importantly, between 28–96 hpf we did not detect a difference in the intensity of GFP fluorescence in the retinas of *rb1* mutant compared to wild type retinas (Figure S1 and data not shown). At 36 hpf wild type RGCs have exited the retina and pioneered across the ventral midline to form an optic chiasm (n = 40, Figure 2A). In contrast, only 13% (n = 30) of *rb1* mutant retinas had RGC axons that exited the retina (Figure 2B, 2C), suggesting that the loss of *rb1* function causes a delay in the initial outgrowth of RGC axons from the retina. At 48 hpf the optic nerve in *rb1* mutants was significantly thinner, with a mean diameter of 3.04 µm (n = 18), compared to the thicker optic nerves in wild type siblings (13.76 µm, n = 22; Figure 2D–2F). At 72 and 96 hpf, when wild type RGC axons have reached and innervated the optic tectum, *rb1* mutant tecta show a significant reduction in RGC axon tectal innervation (Figure 2G–2L; see Methods for quantification details). Together, these results reveal that *rb1* mutants exhibit a delay in RGC axonal outgrowth, leading to a delay in optic nerve development, and reduced innervation of the optic tectum.

### Delayed retinotectal development is caused by a near complete loss of *rb1* function in RGCs

**Figure 3:**
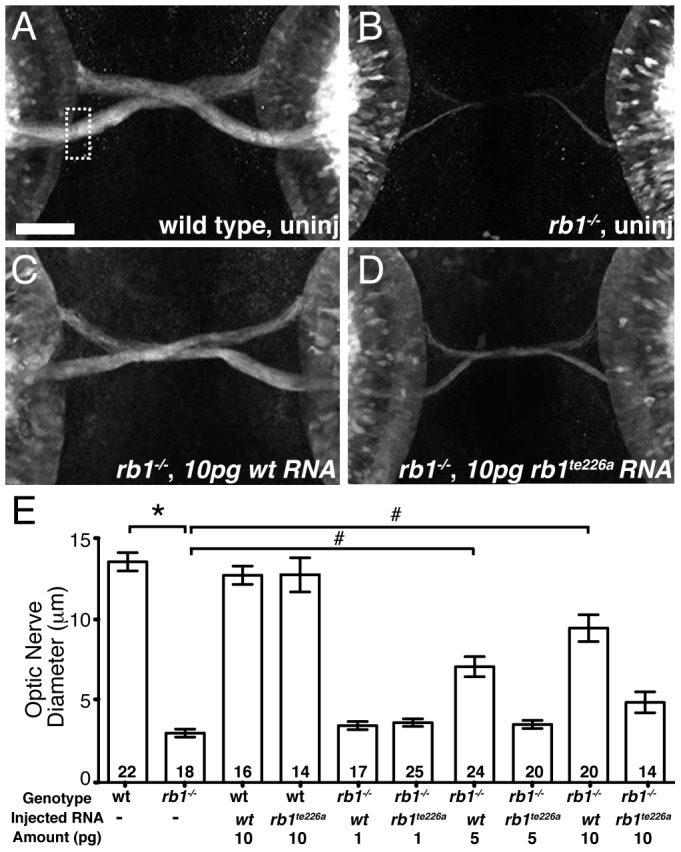
*rb1* mRNA overexpression rescues optic nerve hypoplasia in *rb1^te226^* mutants. (A–B) Optic nerves of uninjected wild type (A) and *rb1^te226a^; ath5:gfp^+/−^* embryos (B) at 48 hpf. (C–D) Optic nerves of *rb1^te226a^; ath5:gfp^+/−^* embryos injected with 10 pg of wild type *rb1* (C) or *rb1^te226a^* (D) mRNA. Dorsal view of maximum intensity projection of confocal z-stacks. (E) Mean optic nerve diameter, measured at dashed rectangle in (A). Error bars denote SEM. *p<0.001, #p<0.01; one-way ANOVA with brackets indicating compared groups. N optic nerves shown at base of bar graphs. Anterior to the top of panel, scale bar = 50 µm.

To determine whether the identified mutation in *rb1^te226a^* is causative of the delay in retinotectal development, we injected wild type *rb1 mRNA* into one-cell stage *rb1^te226a^* mutants and examined optic nerve diameter at 48 hpf. Microinjection of wild type *rb1* mRNA restored optic nerve diameter in *rb1* deficient mutants in a dose dependent manner, demonstrating that mutations in zebrafish *rb1* cause RGC outgrowth defects (Figure 3A–3C, 3E). To determine if and to which extent the mutant *rb1^te226a^* allele has retained biological activity, we examined the ability of *rb1^te226a^ mRNA* to rescue retinotectal development in *rb1^te226a^* mutants (Figure 3D–3E). Injection of *rb1^te226a^ mRNA* failed to significantly increase optic nerve diameter in *rb1^te226a^* mutants, suggesting that the *rb1^te226a^* protein product has very limited, if any, functionality. However, we cannot exclude the possibility that the mutant phenotype is ameliorated by maternal *rb1* mRNA and/or protein deposition.

**Figure 4:**
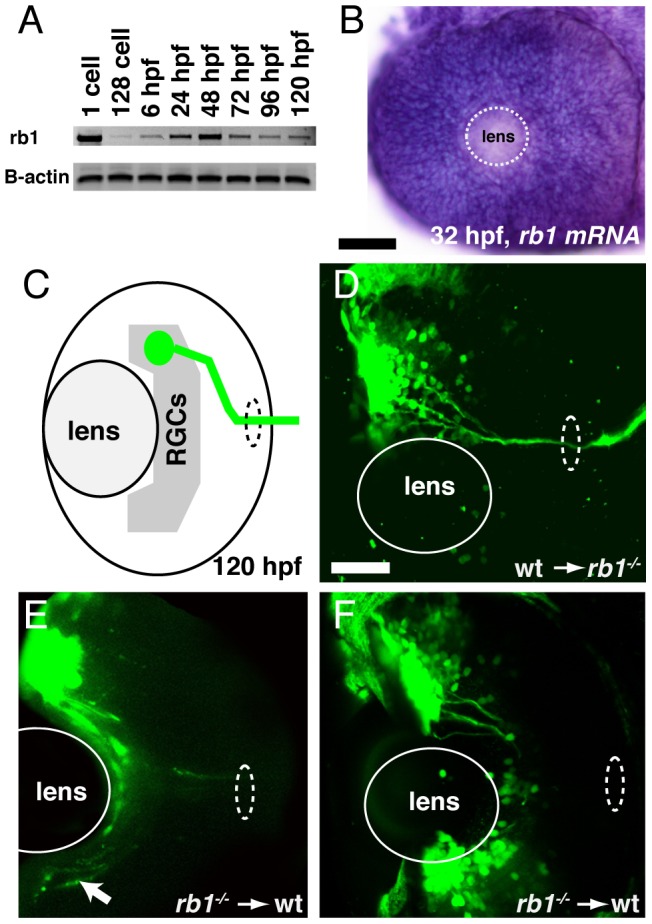
*rb1* is required in RGC axons to regulate retinal exit. (A–B) *rb1* expression by RT-PCR from the 1-cell to 120 hpf larval stage and in the retina by *in situ* hybridization at 32 hpf. B-actin shown as control. White dashed circle marks lens position. (C) Schematic representation of retinal exit by *ath5:gfp* labeled transplanted RGC clones. (D–F) Transplanted *ath5:gfp* labeled clones from wild type donors to *rb1^te226^* mutant retinas (D) and vice versa (E–F). White dashed oval marks retinal exit point. Arrow indicates intraretinally misrouted axons (E). Scale bar = 50 µm.

Finally, we asked whether Rb1 functions within RGCs for their axons to exit from the retina and enter the retinotectal path. Because zebrafish *rb1* is expressed ubiquitously throughout development (Figure 4A–4B), we generated chimeric embryos by transplanting cells at the blastula stage between *rb1* mutant and wild type embryos, and then examined their ability to exit from the retina (Figure 4C). A significant fraction of axons from genotypically mutant *rb1* RGCs transplanted into wild type hosts failed to exit from the retina (19% of retinas showed failure of transplanted *rb1* mutant RGC axons to exit, n = 31, Figure 4E–4F), consistent with the low but significant frequency of *rb1* mutant retinas in which we observed a complete failure of RGCs to exit from the eye (11%; see below). Conversely, 100% of *rb1* mutant retinas showed exit of axons from transplanted wild type RGCs (n = 69, Figure 4D). Thus, during zebrafish development *rb1* acts RGC autonomously for axons to exit the retina and to form the optic nerve.

### Delayed cell cycle exit of *rb1* mutant RGC precursors leads to reduction of “early born” RGCs

**Figure 5:**
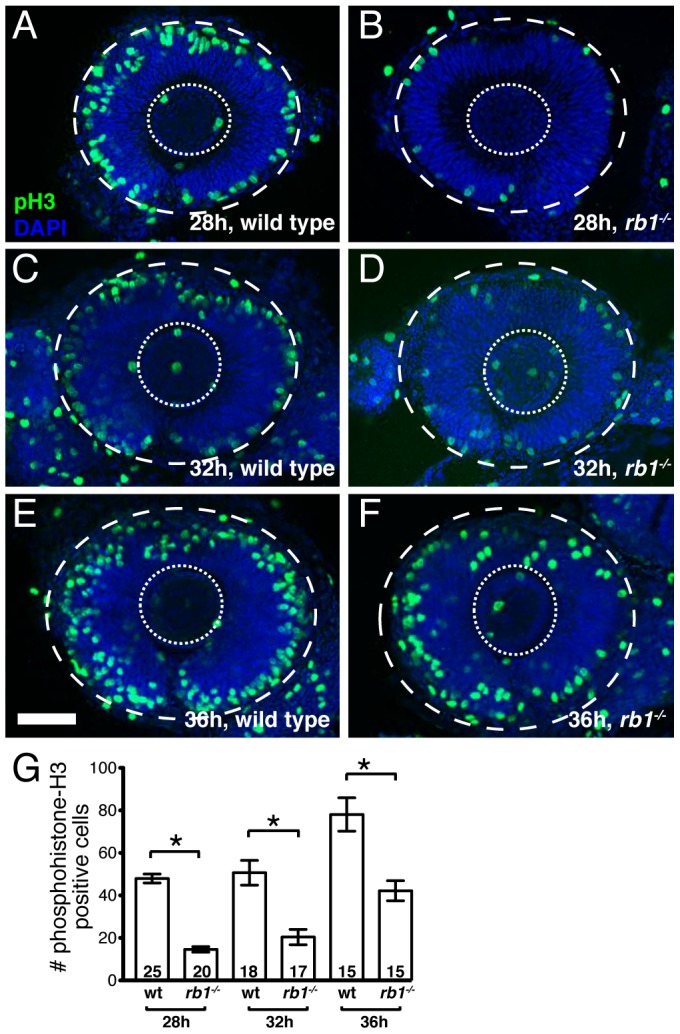
*rb1^te226a^* retinas show delayed cell cycle exit during early retinogenesis. Retinas removed from wild type (A, C, E) or *rb1^te226a^* embryos (B, D, F) at 28 (A–B), 32 (C–D), or 36 hpf (E–F). Retinas labeled with anti-phosphohistone H3 antibody (green) to label M-phase nuclei and counterstained with DAPI (blue). Lateral view of maximum intensity projection of confocal z-stacks. Inner dashed circle outlines lens and outer dashed circle outlines retina. (G) Mean number of anti-phosphohistone-H3 labeled cells per retina. Error bars denote SEM. *p<0.01; one-way ANOVA with brackets indicating compared groups. N retinas shown at base of bar graphs. Anterior to the left, dorsal to the top of each panel. Scale bar = 50 µm.

Given the RGC intrinsic defects observed in *rb1* mutants, we next wanted to determine the primary defect leading to the delay of RGC axons to exit from the retina. Rb1 canonically functions to regulate cell cycle checkpoints, promoting cell cycle exit and differentiation of progenitors and suppressing cell cycle re-entry of differentiated cells [2]. In the retina, *rb1* has been shown to promote the exit of retinal progenitor cells from the cell cycle into the various postmitotic cell types that populate the retinal lamina [9], [10], [11], [12]. To examine *rb1* deficient retinas for cell cycle defects, we labeled wild type and *rb1* mutant retinas for M-phase positive nuclei with an anti-phosphohistone-H3 antibody (anti-pH3) during the initial phase of RGC birth and axon outgrowth, between 28 and 36 hpf. During this time window, premitotic *ath5* positive retinal progenitors divide, with one daughter becoming a postmitotic RGC and the other maintaining its progenitor potency to give rise to other retinal cell types that become postmitotic at later stages of development [21]. Although the total number of M-phase positive increased with time between 28 and 36 hpf in *rb1* mutant and wild type retinas, we observed fewer M-phase positive nuclei in *rb1* deficient retinas, compared to wild type retinas, at each time point examined (Figure 5).

One possibility is that the reduction of M-phase retinal precursors in *rb1* deficient retinas is due to increased cell death. Indeed, compared to wild type retinas, *rb1* deficient retinas showed a slight, but significant increase of TUNEL positive nuclei between 28 and 36 hpf (Figure S1). Importantly though, comparing the increased number of TUNEL positive nuclei to the decreased number of pH3 positive nuclei in *rb1* mutants at 28, 32, and 36 hpf revealed that apoptosis accounts for only 18–26% of the observed reduction in M-phase positive retinal precursors in *rb1* mutant retinas at each time point examined. This suggests that in *rb1* mutant retinas cell death contributes only partially to the deficiency of M-phase positive nuclei (Figure S1G). Thus, the reduction in M-phase retinal precursors in *rb1* mutant retinas suggests a prolonged terminal cell cycle for the retinal precursors, which need to exit their final cycle to become the earliest population of postmitotic RGCs.

**Figure 6:**
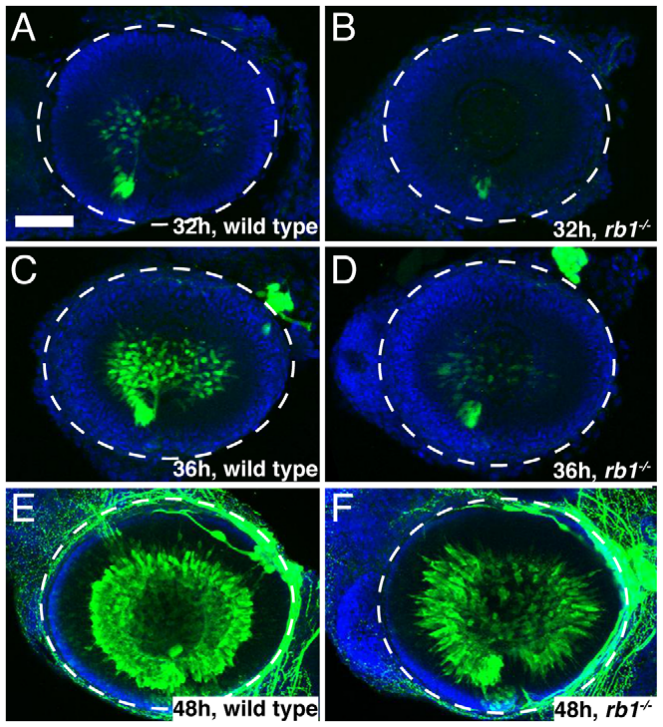
*rb1^te226a^* retinas possess fewer postmitotic RGCs. Retinas removed from wild type (A, C, E) or *rb1^te226a^; isl2b:gfp* embryos (B, D, F) at 32 (A–B), 36 (C–D), or 48 hpf (E–F). Retinas labeled with anti-GFP (green) to label postmitotic, *isl2b* expressing RGCs and counterstained with DAPI (blue). Lateral view of maximum intensity projection of confocal z-stacks. White circle outlines retina. Anterior to the left, dorsal to the top of each panel. Scale bar = 50 µm.

To determine if loss of *rb1* function indeed causes an initial delay in the presence of postmitoic, differentiated RGCs, we examined expression of *isl2b-gfp*, one of the earliest transgenic markers indicative for postmitotic RGCs [22]. We found that in wild type retinas postmitotic, differentiating RGCs marked by *isl2b-gfp* expression emerged first at 32 hpf, increased significantly in their abundance by 36 hpf, and by 48 hpf *isl2b-gfp* positive RGCs were densely packed throughout the ganglion cell layer (Figure 6A, 6C, 6E, n = 25, 16, and 29, respectively). In contrast, *isl2b-gfp* positive RGCs were present in only 13% of *rb1* deficient retinas at 32 hpf (n = 23). Because of the cytoplasmic localization of the GFP signal and the density at which RGCs normally populate the ganglion cell layer, it is difficult to determine the total number of *isl2b-gfp* positive RGCs. Nonetheless, semi-quantitative analysis revealed that by 36 hpf, *isl2b-gfp* positive RGCs were present in 90% of *rb1* mutant retinas (n = 29); however, their distribution with the retina was more similar to that of younger wild type retinas at 32 hpf (Figure 6B, 6D). By 48 hpf, all *rb1* mutant retinas harbored *isl2b-gfp* neurons (n = 32), although differentiation still appeared to lag in 81% (n = 32) of the *rb1* mutant retinas compared to the more densely packed ganglion cell layer in wild type retinas (n = 29, Figure 6E–6F). Despite the reduced number of RGCs present at 48 hpf, *rb1* mutant *isl2b-gfp* positive RGCs express DM-GRASP, a late marker of RGC differentiation, demonstrating that mutant RGCs were fully differentiated once becoming postmitotic (Figure S2). Importantly, the number of *ath5-gfp* positive RGC precursors was unaffected in *rb1* mutants (Figure S1 and data not shown). Thus, the *rb1* deficiency causes a delay in the transition of RGC precursors to postmitotic RGCs, but not in the specification of RGC precursors.

Aside from the reduced population of early born RGCs, *rb1* mutant retinas appear grossly normal, and at 120 hpf, show proper lamination by each retinal cell type [18]. Although we did not determine whether birth dating of other retinal cell types is affected in *rb1* mutant retinas, netrin-positive exit glial cells and Muller glia cells are present in appropriate numbers and location, indistinguishable from wild-type retinas (Figure S2). Taken together, these results suggest that a delay in cell cycle exit by *rb1* deficient RGC precursors leads to a transient reduction in the early born postmitotic RGCs without consequence to the gross morphology and overall cellular landscape of the *rb1* mutant retina.

### *rb1-*deficient RGC axons exhibit intraretinal and midline pathfinding errors

**Figure 7:**
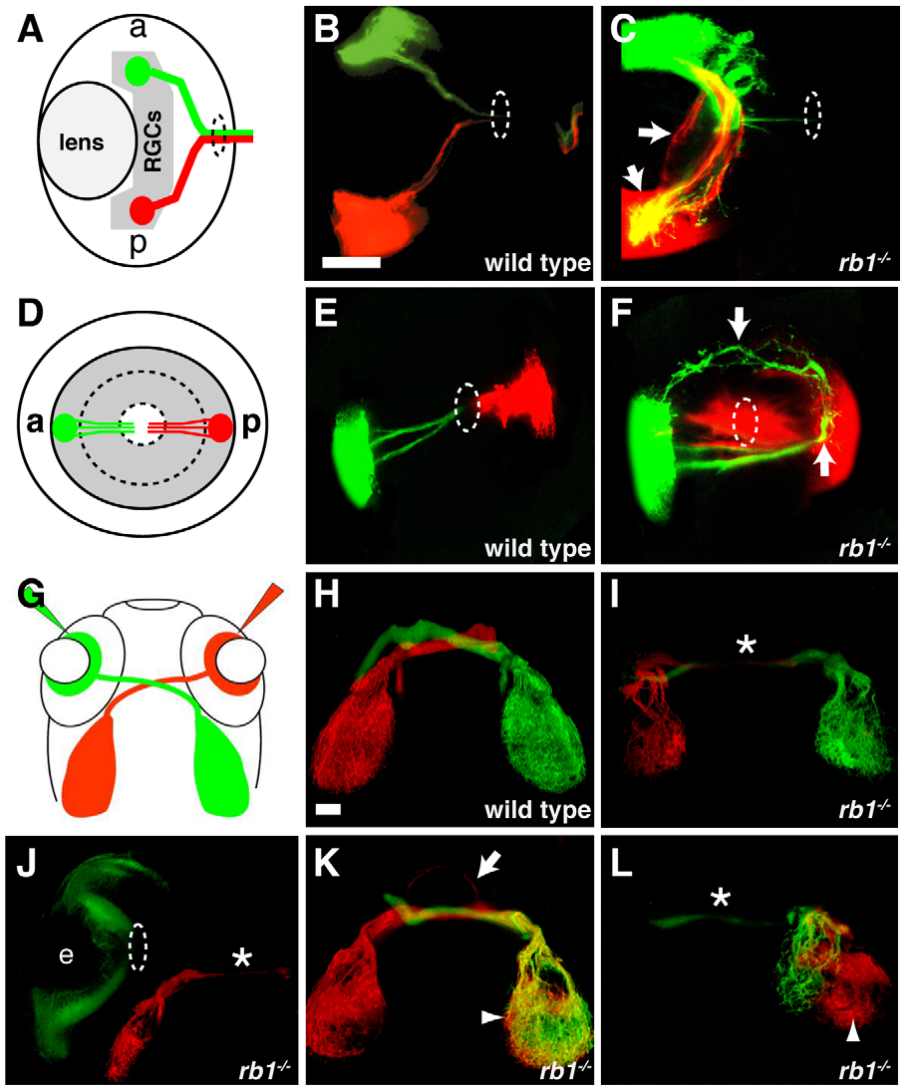
Intraretinal and midline RGC axon pathfinding errors in *rb1^te226a^* mutants. (A–C) Dorsal, intraretinal views of DiO (green) labeled anterior and DiI (red) labeled posterior RGCs. (D–F) Lateral, intraretinal views of DiO/DiI labeled RGCs. (G–L) Dorsal views of retinotectal projection labeled by whole retina fills with DiI/DiO. Eyes removed in H–I, K–L. Dashed circles indicate retinal exit point. Arrows mark misprojecting axons. Asterisk shows optic nerve hypoplasia. Arrowheads mark ipsilateral tectal innervation. Abbreviations: (a) anterior, (p) posterior, (e) eye. Scale bar = 50 µm.

The early born RGCs are located within the central retina and pioneer the retinotectal tract to the contralateral optic tectum [22]. In the absence of the early pioneering RGC axons, the axons of later born, more peripherally located RGCs fail to exit the eye and project aberrantly within the retina [22]. Given the reduced number of these early born, central RGCs in *rb1* mutants, we sought to determine whether peripheral RGC axon trajectories were affected. For this, we labeled small groups of RGCs in the anterior peripheral retina with DiO (green) and in the posterior peripheral retina with DiI (red, Figure 7A–7F). In 120 hpf larvae wild type larvae, all labeled axons from anterior and posterior RGCs fasciculated shortly after sprouting from their soma and extended as an axon bundle, forming a path directly toward the retinal exit point (n = 83, Figure 7A–7B, 7D–7E). In contrast, 91% (n = 65) of *rb1* deficient retinas harbored a significant subset of axons that had extended aberrantly throughout the retina and failed to exit (Figure 7C, 7F). These results demonstrate that the delayed differentiation of the early born RGCs in *rb1* mutants impairs the ability of later born RGC axons to exit the retina.

The delayed cell cycle exit and differentiation of pioneering RGCs lacking *rb1* may also affect axon navigation by later born RGC axons at key choice points: the ventral midline of the diencephalon and/or the optic tectum. To examine these possibilities, we filled the RGC layer of the left and right eyes of wild type and *rb1* mutant larvae with either DiI or DiO, respectively (Figure 7G). In wild type siblings, 99% of dye filled optic nerves projected to their appropriate contralateral tectum (n = 946, Figure 7H). In contrast, *rb1* mutant optic nerves displayed a variety of phenotypes. The majority of *rb1* deficient optic nerves were significantly thinner than their wild type counterparts (37%, n = 663, Figure 7I–7J, 7L), consistent with what we observed in with *ath5:gfp* (Figure 2). In a significant portion of *rb1* deficient optic nerves, 17%, RGCs projected to both the contralateral but also to the ipsilateral tectum, indicative of midline pathfinding defects (n = 663, Figure 7K–7L). Focal DiI/DiO labeling of RGC axons arising from the anterior and posterior retina revealed that retinotopic mapping, a function of retinal cell body location [23], remains intact in *rb1* mutants despite the aberrant pathfinding en route to the optic tectum (Figure S3). Finally, in 11% of *rb1* mutant retinas, there was a complete failure of RGCs to exit from the eye, even at 120 hpf (Figure 7J). Taken together, these results suggest that *rb1* deficient RGC axons make intraretinal and midline pathfinding errors, leading to reduced and incorrect tectal innervation.

### *rb1* mutants display deficits in visually guided behaviors

**Figure 8:**
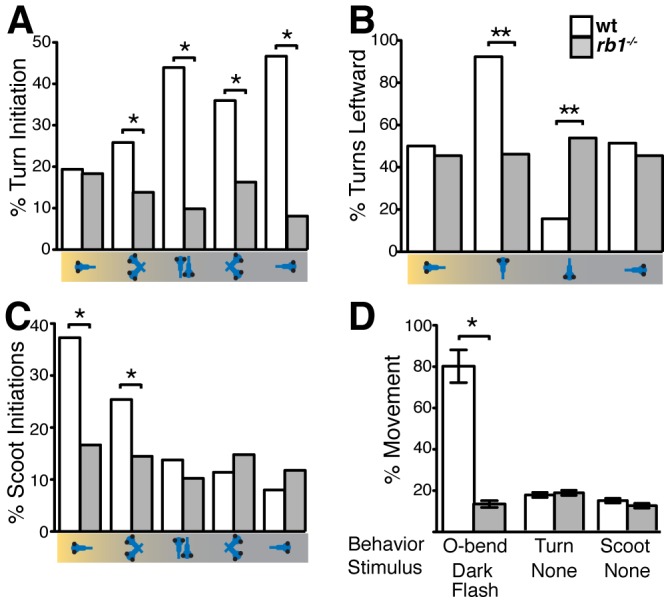
*rb1^te226a^* larvae fail to execute visually guided behaviors. (A) Mean frequency of turns initiated for the first 5 sec of positive phototaxis for subsets of larvae in the schematized orientations with respect to target light (on left). (B) Mean percentage of turns in a leftward direction: 50% represents no directional bias. (C) Mean frequency of scoot swims for the first 5 sec of positive phototaxis for subsets of larvae in the schematized orientations. (D) Mean O-bend response to visual dark flash stimuli and mean initiation of spontaneous turn and scoot swim initiation. Error bars denote SEM. N larvae for each experiment described in Experimental Procedures. *p<0.001, **p<0.01, Wilcoxon-signed rank test. #p<0.001, one-way ANOVA.

By 120 hpf, zebrafish larvae perform an array of sensorimotor behaviors, including responses to visual stimulation. For example, changes in visual field illumination, such as the sudden absence of light or a shift from uniform to focal illumination, elicit specific, stereotyped turning behaviors [24], [25]. We first examined the ability of *rb1* mutant larvae to perform positive phototaxis, defined as navigating toward a target light source that is presented after extinguishing the pre-adapted uniform light field [25]. Positive phototactic navigation is characterized by larvae first turning towards the target light source and then swimming forward towards the target. As previously reported, when presented with a target light source wild type larvae facing away from the light target show significant initiation of turns, which are preferentially biased towards the light target (Figure 8A–8B). Once facing the target, wild type larvae initiate forward scoot swims (Figure 8C). In contrast, turn initiation in *rb1* mutant larvae facing away from the light target was dramatically reduced (Figure 8A). On the few occasions when they initiated a turn, turning direction was unbiased with respect to the light target (Figure 8B). Moreover, *rb1* mutants facing the light target did not show an increase in forward scoot swim initiation above baseline (Figure 8C). To further determine whether *rb1* mutants respond to more extreme changes in illumination, we examined their ability to perform an O-bend response to a visual dark flash stimulus, a sudden extinction of light [24]. Again, compared to their wild type siblings, *rb1* mutants displayed a minimal O-bend response to dark flash stimulation (Figure 8D). Despite their impaired visual responses, *rb1* mutants showed no difference in the spontaneous initiation of turning or swimming behaviors compared to wild type siblings (Figure 8D). Importantly, the kinematic parameters of spontaneously occurring turning and swimming movements were indistinguishable between *rb1* mutants and their wild type siblings, demonstrating that the neural circuits required for initiation and execution of turning behaviors are largely intact in *rb1* mutants. Together, these results demonstrate that *rb1* mutants exhibit visual deficits.

## Discussion

Children with biallelic germline or sporadic inactivation of *rb1* are likely to form ocular tumors during early childhood. Initially, the retinas of affected individual show an otherwise grossly normal morphology. In contrast, even conditional *rb1* knockout mouse models exhibit ectopic proliferation and cell death leading to significant morphological defects throughout affected retinas. We find that inactivation of the zebrafish *rb1* gene through a *rb1* causing mutation results in mutant retinas that display very limited signs of cell death, with differentiated retinal cell types that are properly laminated, similarly to childhood retinas lacking *rb1*. Thus, the fairly ‘normal’ retinal landscape of zebrafish *rb1^te226a^* mutants provided us with a unique opportunity to investigate if and how *rb1* is required to establish the retinotectal projection. Our analysis reveals a RGC autonomous requirement for *rb1* in regulating RGC axon pathfinding within the retina and at presumptive choice points en route to the optic tectum. Moreover, we demonstrate that zebrafish *rb1^te226a^* mutants exhibit deficits in visually guided behaviors, suggesting that the retinotectal path defects in *rb1* mutants may be sufficient to impair vision. Together, this work reveals a novel role for *rb1* in the establishment of RGC axon projections during development and establishes a unique model for understanding the developmental and tumor suppressor roles of the *rb1* gene.

Zebrafish *rb1^te226a^* mutants harbor a human retinoblastoma causing *rb1* gene mutation. The mutant protein is truncated in the B-domain and lacks the cyclin-binding domain, reducing Rb1's capacity to form a ‘pocket’, and reducing its capacity for phosphorylation by cyclin dependent kinases [2], [26]. Consistent with the notion that the *rb1^te226a^* mutant allele is largely non-functional, mRNA over-expression in *rb1* mutants does not ameliorate the *rb1* mutant phenotype. Despite the absence of biological activity of the truncated *rb1^te226a^* protein, mutant zebrafish show a significantly milder retinal phenotype compared to conditional or even germline *rb1* mouse knockouts [6], [7], [8], [9], [10], [11], [12]. One possible explanation is the strong maternal contribution of *rb1* in zebrafish (Figure 4A), which may suppress phenotypic expressivity at early stages of development. Consistent with this idea, formation of the initial scaffold of axon tracts during the first day of development appears unaffected in *rb1^te226a^* mutants, yet visual and hindbrain pathways that develop after the first day of development show defects [18].

In humans the *rb1* nt1960+1 mutation, which is identical to the zebrafish *rb1^te226a^* mutation, causes ocular tumors [13], [14], [15], [16], raising the possibility that zebrafish *rb1* mutants might also develop tumors as juveniles. However, *rb1* mutants fail to inflate a functional swim bladder, and die ∼7 days post fertilization, precluding the analysis of ocular tumors in juveniles. Although wild type *rb1* mRNA injection rescues the early RGC and retinotectal defects through 48 hpf, these transiently rescued *rb1^te226a^* mutants do not survive beyond 7 days of development, indicating that *rb1* plays a critical role after the injected mRNA has been degraded. Establishing stable, inducible *rb1* transgenic lines to rescue developmental deficits will therefore be required to monitor juvenile and adult zebrafish for retinal tumors.

In *rb1^te226a^* mutants, a significant subset of RGC axons fail to exit the retina, and many of the exiting axons then project incorrectly to the ipsilateral tectum, revealing a previously unrecognized requirement for *rb1* in regulating axon pathfinding. One possible explanation for the RGC guidance defects is that in *rb1^te226a^* mutant RGCs the expression of guidance factors might be disrupted. Interestingly, cortical cell migration was shown to be dependent on *rb1* regulated neogenin expression [27], suggesting that *rb1* deficient RGC axons might lack guidance factors required to navigate towards the retinal exit point and properly cross the ventral midline. To investigate this possibility, we performed microarray gene expression analysis of the retina and brains of 32 hpf *rb1^te226a^* mutants (MAW and MG, unpublished). However, this approach did not reveal a significant change in the expression levels of neogenin or other known axon guidance genes. Although it remains possible that *rb1* regulates expression of un-identified guidance factors, it is more likely that *rb1* regulates axon pathfinding indirectly by ensuring the timely exit of RGC precursors from the cell cycle and hence the appropriate temporal appearance of differentiated RGCs. In fact, genetic ablation of the earliest born RGCs prevents the formation of the retinotectal tract [22]. This suggests that RGC birth order imprints a critical hierarchical pathfinding role on RGC axons, such that axons from the earliest born RGCs pioneer the retinotectal tract that later born RGC axons will follow [22]. The delayed onset of RGC birth in *rb1^te226a^* mutants may therefore reduce the population of pioneering RGCs present during a restricted window of environmentally expressed guidance factors.

Zebrafish *rb1^te226a^* mutants display deficits in the acoustic startle response [17], [18] and in visually guided behaviors, reflecting the importance of *rb1* function for the development of neural circuitry underlying behavior. The deficits in startle behavior are due to defects in a small subset of hindbrain neurons, the spiral fiber neurons [18], and giving the results presented here, it is tempting to speculate that *rb1* plays a similar role for the transition of these neurons from precursors to postmitotic neurons. Unfortunately, markers that follow the development of spiral fiber neurons are not available, precluding such analysis. Therefore, we focused on the well-characterized development of RGCs. Although we demonstrate a defect in the early development of these cells and their axonal connectivity, we cannot exclude the possibility that zebrafish *rb1* mutants exhibit defects in the development and/or function of other retinal cell types, and that these defects contribute to the deficits in visual behaviors we observe. Future analysis of transgenic lines expressing the wild type *Rb1* gene in individual retinal cell types will reveal which cell type(s) and connections are causative of the visual deficit.

In summary, we report a zebrafish mutant carrying a human disease causing *rb1* mutation, which reveals novel roles of *rb1* in regulating RGC axon pathfinding and visually guided motor behavior. Furthermore, these mutants provide a non-murine vertebrate model of *rb1* and offer new potential for identifying the elusive retinoblastoma cell of origin and further insight into the developmental role of *rb1.*

## Materials and Methods

### Ethics statement

All experiments were conducted according to an Animal Protocol fully approved by the University of Pennsylvania Institutional Animal Care and Use Committee (IACUC) on January 27, 2011, protocol number 803446. Veterinary care is under the supervision of the University Laboratory Animal Resources (ULAR) of the University of Pennsylvania.

### Animals and fish maintenance

The zebrafish (*Danio rerio*) strain used in this study was the *spc^te226a^* allele (now referred to as *rb1^te226a^*) of *space cadet* [17], [18], maintained on a mixed TLF and Tubingen background. The *rb1^te226a^* allele was also crossed into the *ath5:gfp* and *isl2b:gfp* transgenic backgrounds for RGC analysis [20], [22]. *rb1^te226a+/−^;ath5:gfp^+/−^* or *rb1^te226a+/−^;isl2b:gfp^+/−^* adults were always crossed with *rb1^te226a+/−^;TLF* adults to ensure that *rb1^te226a^* embryos analyzed for GFP-expressing RGCs were hemizygous for GFP. Throughout the manuscript, *rb1^−/−^*, “*rb1* deficient”, and *rb1* mutant refers to *rb1^te226a^* homozygotes. The other *space cadet* allele *spc^ty85d^* [17] was only used where mentioned. Embryos were collected in the morning, maintained on a 14/10 hour light/dark cycle at 28°C, and staged as described previously [28]. Larvae were raised in 6 cm plastic Petri dishes at a density of 20–30 per 7 mL in E3 medium (5 mM NaCl, 0.17 m mM KCl, 0.33 mM CaCl_2_, 0.33 mM MgSO_4_) with medium changes at 48 hpf (hours post fertilization) and 96 hpf. Behavioral experiments were conducted on 120 hpf larvae.

### Recombination mapping and molecular cloning of *rb1*

A three generation mapping cross between *rb1^te226a^* heterozygous and WIK fish was generated, and pools of 25 F_2_ mutant and F_2_ sibling 5 dpf larvae were collected in the F_2_ generation and used for bulk segregant mapping (see Table 2 for simple sequence length and single nucleotide polymorphic markers). Mutant larvae were identified by performing successive, unilateral C-bends to acoustic or tactile stimulation [17], [18]. To identify the mutation, cDNA was prepared following total mRNA extraction from 5 dpf larvae as previously described [29]. *rb1* cDNA was amplified with primers (*rb1*:1–6, Table 2) designed against overlapping regions of the *rb1* reference sequence (Ensembl) with the following RT-PCR conditions: 94°C for 3 min and then 40 cycles of 94°C for 45 sec, 57°C for 1 min, and 70°C for 1 min. Products were gel purified and cloned into the pCR2.1-TOPO-TA vector for sequencing. After detecting a frameshift and 4 nucleotide addition to the end of exon 19 in *rb1^te226a^* cDNA clones, gDNA was isolated from 5 dpf larvae, and intron 19 was amplified with the *rb1*:8 primers, using identical PCR conditions to those described above.

**Table 2:** PCR primers for recombination mapping, molecular cloning, and genotyping of *rb1^te226a^*.

Primer	Forward	Reverse
*cDNA amplification*		
*rb1-*1 (180)	CCGACTACCAACCACTAACC	CTAACCACCGAATTGTAGGG
*rb1-*2 (181)	ACAATTCGGTGGTTAGTTCC	TAAGGAGCTCCCAACATAGG
*rb1-*3 (211)	GCAGCGTTTCATACATCATTGC	GGTCAGCAGATTGGAGAACAGC
*rb1-*4 (183)	GGACTCTCCACTGTTTGACC	CTTGAGTGACCAGTCAGAGC
*rb1-*5 (184)	TCAATACGACTCCATCATCG	GGTATGTTTCACGTCAACTCC
*rb1-*6 (208)	AACAGCACAGATGCTTTCC	CATGTCAGTTGTTAAACTTTCG
*rb1-*FL (Rb1-4)	AAACATCACAACACAACTCG	CCCAGTAATGCTTAAACACC
B-actin	TACAGCTTCACCACCACAGC	AAGGAAGGCTGGAAGAGAGC
*gDNA amplification*		
*rb1-8* (E19-I19) splice site	GTGAGTGTGAGTGTGAGACC	ACTGTACCTAGTGTGAGTACGG
Z20205 SSLP	ACGGCATCTCAGTGTGACAG	CACGCTACAGGAGCAAAACA
Z11343 SSLP	GATCTTTCAGCTTTGGCTGC	CAAACACTGAGCTTCCCTCC
NCAM1 SNP	GCACACAGTCTCCTTTGGACAG	GATAGTTCACTGGCTCCAAACCC
Myo5b SNP intron 6–7	CAATGTTCGGCCTGCCAAACCC	AACATAATGAGGGGTGGTTGC
*rb1-*dCAPS w/SspI	CCAGGTATCTCTCTCCTGTCC	GCATGTGAATGTAAACTACGGTTTAACCAA

For *rb1* RNA injection, cDNA was prepared from genotyped homozygous wild type or *rb1^te226a^* mutant 5 dpf larvae (dCAPS protocol, see below) and amplified with the *rb1*:FL primers (similar conditions as above, except extension time increased to 3 min), which includes the coding region of *rb1,* and cloned into the pCS2^+^ vector. Wild type *rb1* and *rb1^te226a^* mRNA was prepared using the mMessage mMachine kit (Ambion, NY) and injected at the 1-cell stage at doses ranging from 1–100 picograms. Embryos injected with 20 or greater picograms of *rb1* mRNA showed gross morphological abnormalities and necrosis, whereas embryos injected with 10 picograms or less appeared morphologically normal.

### PCR genotyping *rb1^te226a^*

To genotype *rb1^te226a^* embryos, we developed a dCAPS assay [30] using the dCAPS program (http://helix.wustl.edu/dcaps/dcaps.html) to design appropriate primers (Table 2). After gDNA isolation, PCR was performed as described above. The PCR product is then digested with SspI (New England Biolabs, Ipswich, MA), cleaving the *rb1^te226a^* allele and producing a 120 bp fragment that can be distinguished from the 150 bp wild type allele on a 3% agarose gel containing 1.5% Metaphor agarose (Lonza, Rockland, ME). All genotyping, except for BrdU labeled embryos, was performed following immunolabeling experiments.

### Immunohistochemistry and *in situ* hybridization

For immunostaining, embryos were fixed in 4% paraformaldehyde (PFA) overnight at 4°C, permeabilized with 1 mg/mL collagenase, and blocked for 1 hour with 5% normal goat serum in 0.1 M phosphate buffer. Embryos were then incubated in the primary antibodies anti-GFP (1∶200 mouse JL8, Clontech, Mountain View, CA or 1∶500 rabbit, Invitrogen, Carlsbad, CA), anti-phosphohistone-H3 (Millipore, Charlottesville, VA), 1∶100 anti-BrdU (Roche, Branchburg, NJ), and/or 1∶50 A2-J-22 polyclonal antisera (recognizes carbonic anhydrase II, kindly provided by Dr. P. Linser) overnight at 4°C in blocking solution, washed out, and then detected by the addition of AlexaFluor488 or AlexaFluor594 conjugated secondary antibodies (1∶500, Invitrogen, Carlsbad, CA). TUNEL assay was performed as previously described [31] using Apoptag Peroxidase In Situ Apoptosis Detection Kit (Chemicon, Temecula, CA). After staining, samples were mounted in DAPI containing Vectashield (Vector Labs, Burlingame, CA). Images were acquired with a Zeiss 710 confocal laser scanning microscope (LSM 710) using ZEN2010 software.

For *in situ* hybridization, digoxygenin-UTP labeled antisense riboprobes for *rb1* were synthesized and hydrolyzed from the full length *rb1* cDNA construct [32]. Whole-mount *in situ* hybridization was performed as described previously [33]. Images were acquired with a Zeiss Axioskop compound microscope. For RT-PCR based expression analysis, the *rb1:FL* and *B-actin* primers (Table 2) were run against cDNA prepared from total mRNA extracted from 25 embryos/larva at each stage.

### Lipophilic dye labeling of retinal ganglion cells

120 hpf larvae were anesthetized (0.01% Tricaine) and fixed in 4% paraformaldehyde at 4°C overnight. Larvae were removed from fix, washed briefly in phosphate buffered saline (PBS), and mounted dorsal side up for whole retinal injection or laterally for discreet RGC labeling on glass microscope slides in a bed of 1.5% agarose. To label all RGCs, the vitreal space of each eye was filled with either of the fluorescent lipophilic dyes DiI (red) or DiO (green) (Molecular Probes, Eugene, OR) dissolved in 1% chloroform, using a WPI PV820 picopump injector fitted with a glass micropipette. For discreet labeling, a small region of the exposed eye was labeled with pulses of DiI/DiO dissolved in 0.5% dimethylformamide. Injected larvae were kept moist with PBS and incubated overnight at room temperature in a humidity chamber in darkness. Larvae were then examined for phenotype analysis using a Zeiss Axioplan compound fluorescent microscope. Eyes were carefully removed from selected representative larvae, which were then remounted on coverslips in agarose for imaging. Images were recorded using a Zeiss 510 confocal laser scanning microscope (LSM510) and Zeiss LSM510 analytic software.

### Cell transplantation

For transplant direction wild type donor into *space cadet* host, wild type transgenic *Tg(ath5:gfp)* and *rb1^te226a^* heterozygous fish were used to generate wild type GFP expressing donor embryos and non-GFP expressing *rb1^te226a^* mutant embryos, respectively. For transplant direction space cadet donor into wild type host, *rb1^te226a^*; *Tg(ath5:gfp)* double heterozygotes and either TU or TLF strain wild type mating pairs were used to generate *rb1^te226^* GFP expressing donor embryos and non-GFP expressing wild type embryos, respectively. Once the appropriate donor-host embryos were collected, embryos were immediately placed in E3 medium and kept at room temperature. Donor embryos were pressure injected into the yolk sac at the 1–2 cell stage with the lineage tracer tetramethylrhodamine dextran, 3 Kd, 5% w/v (Molecular Probes, Eugene, OR) dissolved in 0.2 M KCL and filter sterilized. Donor and host embryos were then incubated at 28.5°C in E3 medium in darkness to grow synchronously to the 1000 cell stage. Embryos were then transferred into room temperature complete E2 medium (E2) to retard growth, and dechorionated using Pronase (1∶50 in E2 of 30 mg/ml stock, Roche) in glass 60 mm petri dishes. Dechorionated embryos were washed extensively with E2, transferred using a fire polished glass Pasteur pipette into individual wells in a transplantation dish containing E2, and properly oriented. Transplantation needles were made using #1BBL No Fil borosilicate glass pipettes (WPI), pulled to produce fine tips in a P87 pipette puller (Sutter Instruments, Novato, CA), broken at various diameter openings, and polished using a microforge. Needles were then inserted into a standard pipette holder connected to a modified manual injection apparatus, and mounted in a micromanipulator arm for precision control. Thirty to fifty blastomeres were carefully removed from the donor embryo using the transplantation pipette/manual injector apparatus, and transferred into the adjacent host embryo at the apex of the animal pole (eye/nose region). Operated embryos were maintained in the transplantation dish wells in E2 at 28.5°C in darkness following transplantation, and were allowed to develop undisturbed until epiboly completed. Embryos were then transferred from the transplantation wells/dish into either separate 1.5% agarose coated 60 mm plastic Petri dishes for donors and hosts, or 1.5% agarose coated wells in 12-well tissue culture plates as host-donor pairs, depending on the direction of the transplant, and incubated at 28.5°C for five days. The later was necessary in order to correctly identify *rb1^te226a^* donors from each donor-host pair, as the motility phenotype does not manifest itself until 120 hpf. 120 hpf larvae were screened for the presence of GFP expressing RGC clones using a Leica MZFIII fluorescence stereomicroscope, and further analyzed for misprojecting RGC axons using a Zeiss axioplan compound fluorescence microscope. Host larvae suspected of containing misprojecting RGC axons (ie, not exiting the eye, or midline defects) were then fixed and stained with anti-GFP antibody as described above, and imaged using a Zeiss LSM510 microscope and Zeiss LSM510 analytic software. Confocal z-stacks were of sufficient depth (150–220 µm) to insure optic nerves were not inadvertently missed.

### Image analysis and quantification

Confocal stacks were processed into maximum and/or summation intensity projections using ImageJ for quantification. We used the full width half maximum algorithm to calculate optic nerve diameter from maximum intensity projections of GFP-labeled retinal ganglion cell axons. Tectal innervation was determined by making 20 µm summation projections of GFP labeled tecta, tracing the area of the labeled tectum to determine the Raw Integrated Density (RID) per µm^2^, and subtracting the RID/µm^2^ of an unlabeled, background region. TUNEL and anti-pH3 labeled nuclei were counted from 30 µm stacks using Volocity (PerkinElmer, Waltham, MA), with individual cells distinguished by fluorescent intensity and size. Statistical analysis was performed on all data using the Graphpad prism software (www.graphpad.com).

### Behavioral analysis

Behavioral experiments were performed on 120 hpf larvae and analyzed with the FLOTE software package as previously described [25], [34], [35]. *rb1^te226a^* and wild type siblings were identified based on acoustic startle behavior [17], [18] and then grouped by phenotype for visual behavior testing in 6 cM petri dishes at a density of 12 fish per dish. For all behavioral experiments, N = 48 *rb1^te226a^* and 48 wild type sibling larvae. For phototaxis experiments, video recordings were triggered every 500 msec, with each recording covering a 400 msec time window, for a total duration of 4 sec of recorded behavior. Each group of 12 larva were subjected to 3 rounds of phototaxis testing, with 3 min between trials. Orientation of larvae to target light was determined at the beginning of each 400 ms recording as previously described [25], such that the behavior of each larva was tested multiple times and in different orientations with respect to the target light. Therefore, the N for Figure 8A and 8C ranged from 75 to 547 for wild type siblings and 136 to 311 for *rb1^te226a^* larvae. In Figure 8B, the N ranged from 39 to 106 for wild type siblings and 13–33 for *rb1^te226a^* larvae. For dark flash response experiments, N = 4 groups of 12 larvae. Spontaneous behavior was analyzed on individually housed larvae on a 4×4 grid array.

## Supporting Information

Figure S1 *rb1^te226a^* retinas show increased apoptosis. Retinas removed from wild type (A, C, E) or *rb1^te226a^; ath5:gfp* embryos (B, D, F) at 28 (A–B), 32 (C–D), or 36 hpf (E–F). Retinas labeled anti-GFP (green), TUNEL (red), and counterstained with DAPI (blue). Lateral view of maximum intensity projection of confocal z-stacks. White dashed circle outlines retina. Anterior to the left, dorsal to the top of each panel. (G) Mean number of TUNEL positive nuclei per retina. Error bars denote SEM. *p<0.01; one-way ANOVA. N retinas shown at base of bar graphs. Scale bar = 50 µm.
(TIF)

Figure S2 *rb1^te226a^* retinas express markers indicating normal gross morphology. Retinas removed from wild type (A, C, E) or *rb1^te226a^* embryos (B, D, F) at 36 (A–B), 48 (C–D), or 120 hpf (E–F). (A,B) *netrin in situ* shows presence of glial cells at optic stalk of retina. (C, D) DM-GRASP immunolabeling labels postmitotic, differentiated RGCs. White dashed circle outlines retina. (E, F) Carbonic anhydrase II (CA II) immunolabeling identifies Muller glia cell bodies (arrows) and endfeet (arrowheads). Lateral views of maximum intensity projection of confocal z-stacks. Anterior to the left, dorsal to the top of each panel. Scale bar = 50 µm.
(TIF)

Figure S3 Retinotopic mapping is intact in *rb1^te226a^* tectum. Dorsal views of schematized (A, C) and confocal projections (B, D) of retinotectal projection in wild type (A, B) and *rb1^te226a^* larvae at 120 hpf. DiO (green) labeled axons from anterior RGCs innervate the caudal tectum, while DiI (red) labeled axons of posterior RGCs project to the rostral tectum in both wild type and *rb1^te226a^* larvae. Arrowheads mark misprojecting axons. Scale bar = 50 µm.
(TIF)
