# Supplementary figures and images for: The Tumor Suppressor Gene Retinoblastoma-1 Is Required for Retinotectal Development and Visual Function in Zebrafish
Source: PLoS Genet. 2012 Nov 29;8(11):e1003106. doi: 10.1371/journal.pgen.1003106 (PMC3510048; doi:10.1371/journal.pgen.1003106)

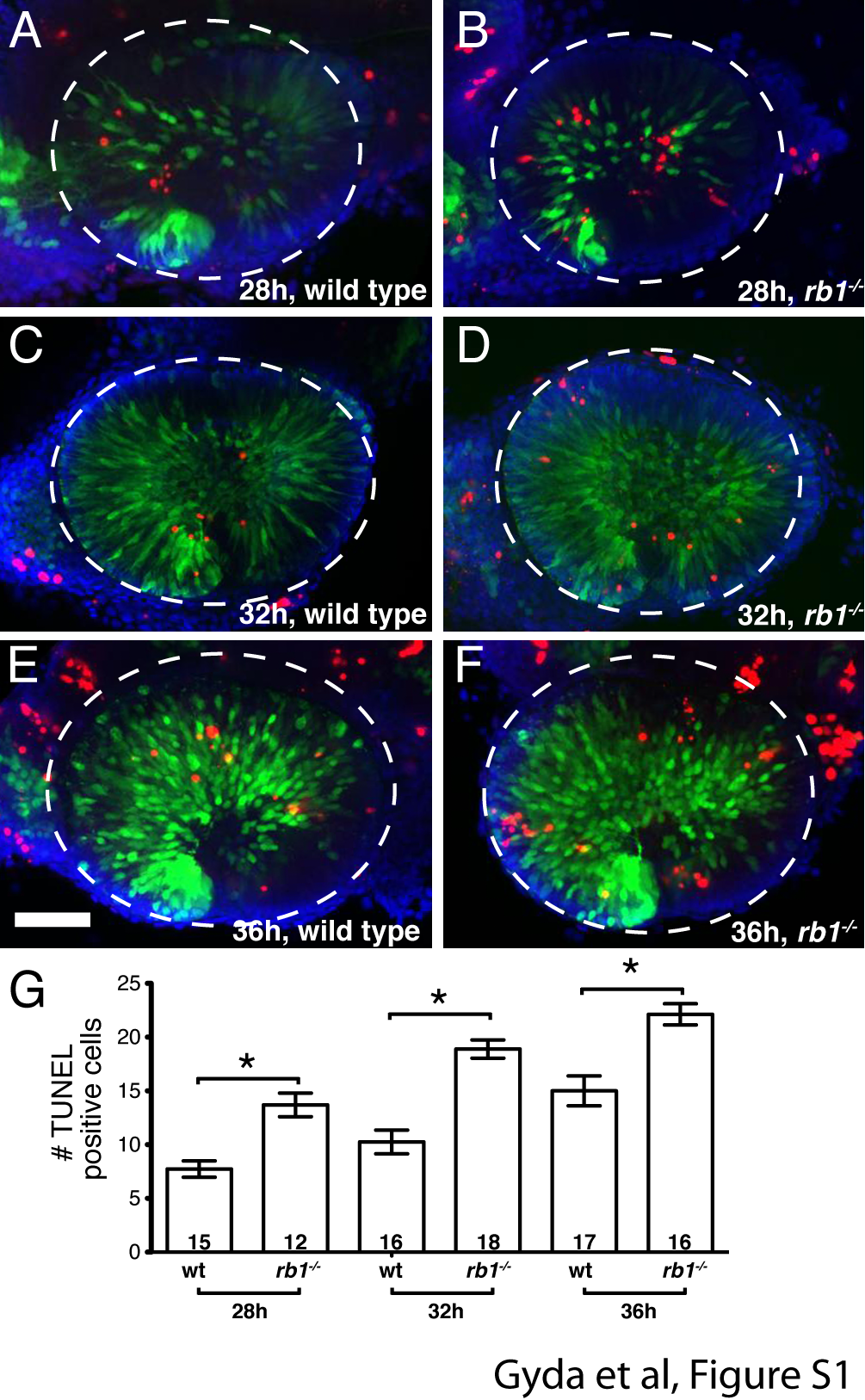

Supplement: Figure S1 — rb1te226a retinas show increased apoptosis. Retinas removed from wild type (A, C, E) or rb1te226a; ath5:gfp embryos (B, D, F) at 28 (A–B), 32 (C–D), or 36 hpf (E–F). Retinas labeled anti-GFP (green), TUNEL (red), and counterstained with DAPI (blue). Lateral view of maximum intensity projection of confocal z-stacks. White dashed circle outlines retina. Anterior to the left, dorsal to the top of each panel. (G) Mean number of TUNEL positive nuclei per retina. Error bars denote SEM. *p<0.01; one-way ANOVA. N retinas shown at base of bar graphs. Scale bar = 50 µm. (TIF) [file pgen.1003106.s001.tif]

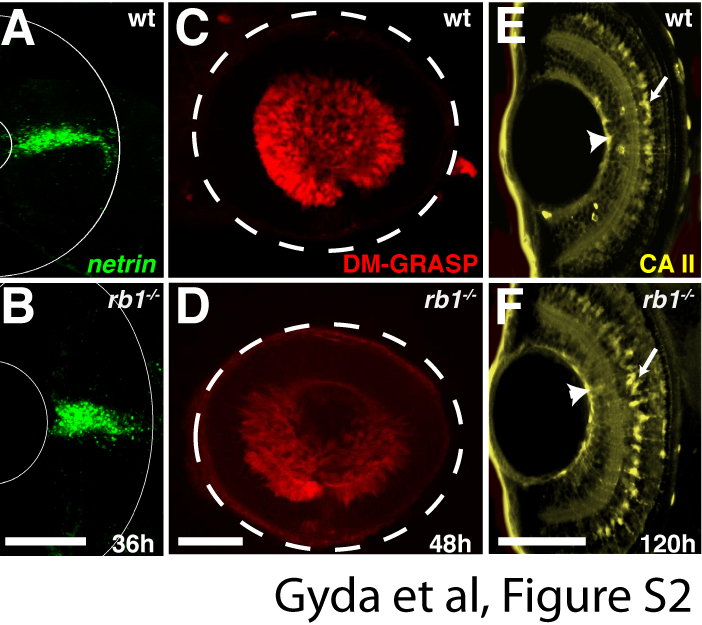

Supplement: Figure S2 — rb1te226a retinas express markers indicating normal gross morphology. Retinas removed from wild type (A, C, E) or rb1te226a embryos (B, D, F) at 36 (A–B), 48 (C–D), or 120 hpf (E–F). (A,B) netrin in situ shows presence of glial cells at optic stalk of retina. (C, D) DM-GRASP immunolabeling labels postmitotic, differentiated RGCs. White dashed circle outlines retina. (E, F) Carbonic anhydrase II (CA II) immunolabeling identifies Muller glia cell bodies (arrows) and endfeet (arrowheads). Lateral views of maximum intensity projection of confocal z-stacks. Anterior to the left, dorsal to the top of each panel. Scale bar = 50 µm. (TIF) [file pgen.1003106.s002.tif]

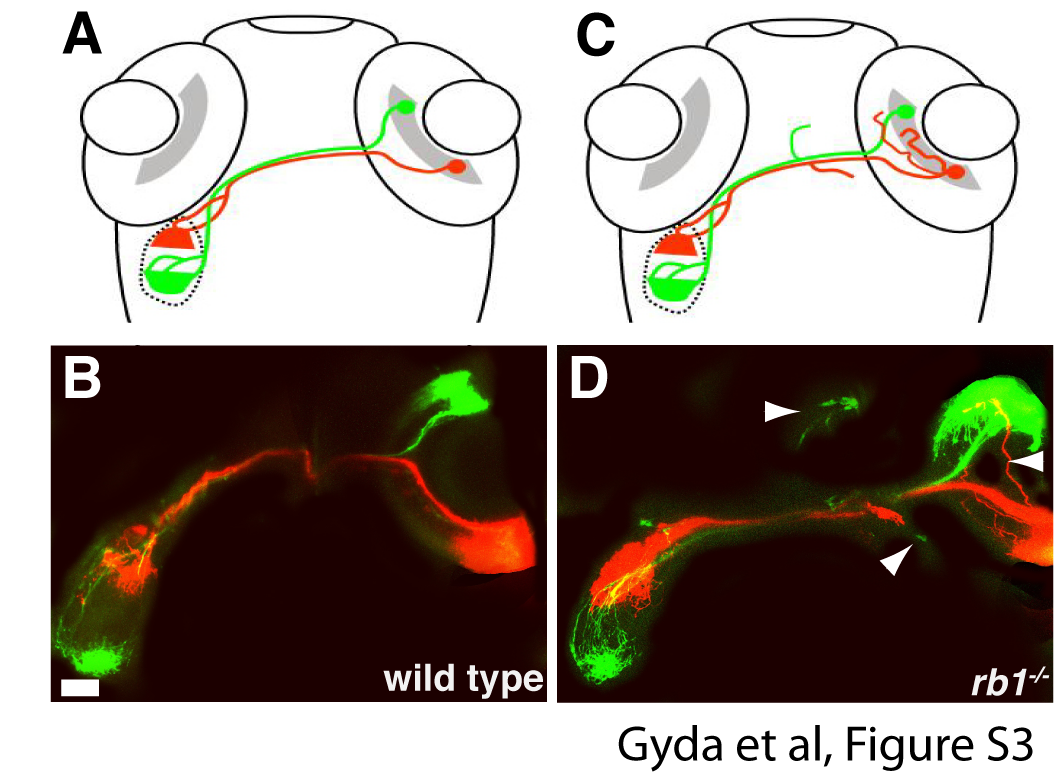

Supplement: Figure S3 — Retinotopic mapping is intact in rb1te226a tectum. Dorsal views of schematized (A, C) and confocal projections (B, D) of retinotectal projection in wild type (A, B) and rb1te226a larvae at 120 hpf. DiO (green) labeled axons from anterior RGCs innervate the caudal tectum, while DiI (red) labeled axons of posterior RGCs project to the rostral tectum in both wild type and rb1te226a larvae. Arrowheads mark misprojecting axons. Scale bar = 50 µm. (TIF) [file pgen.1003106.s003.tif]
